# Supplementary material for: Near-atomic structure of a giant virus
Source: Nat Commun. 2019 Jan 23;10:388. doi: 10.1038/s41467-019-08319-6 (PMC6344570; doi:10.1038/s41467-019-08319-6)
Supplement: Supplementary file 1 — Supplementary information [file 41467_2019_8319_MOESM1_ESM.docx]

Near-atomic structure of a giant virus

Q. Fang, D. Zhu et al.

**Supplementary** **Information**


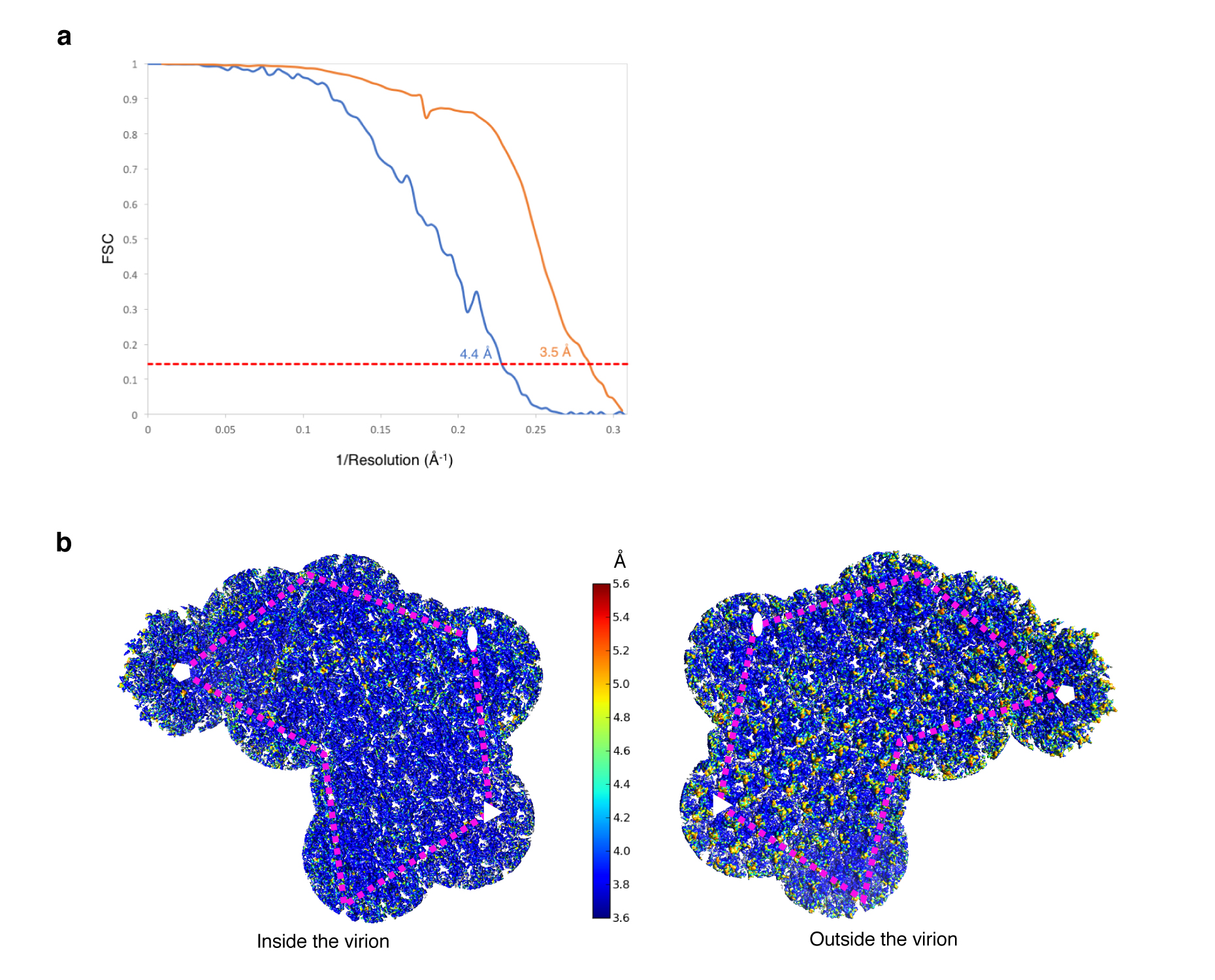


Supplementary Figure 1. Resolution estimation of the cryo-EM maps. a Gold-standard FSC curves for the 3D cryo-EM reconstructions of the icosahedrally averaged PBCV-1 capsid. The blue colored curve relates to the original 4.4 Å resolution map obtained by conventional reconstruction techniques^1^. The orange colored curve relates to the improved 3.5 Å resolution map using techniques as previously described^2^. b Local resolution within one asymmetric unit of the final icosahedrally averaged PBCV-1 map as estimated using the RESMAP program^3^. The boundaries of the asymmetric unit are outlined in dashed magenta lines. The icosahedral 5-, 3- and 2-fold axes are shown as white pentagons, triangles and ovals, respectively.


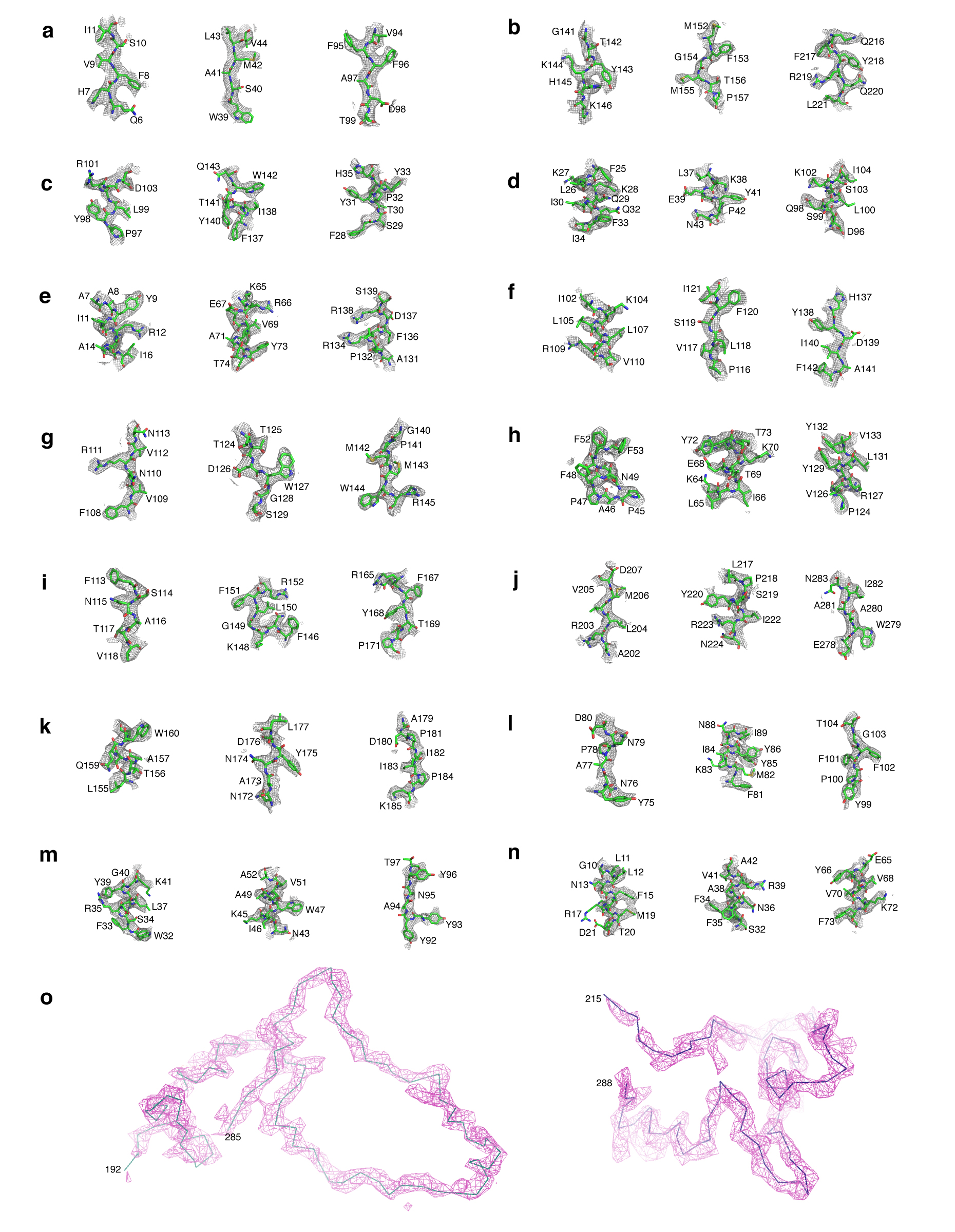


Supplementary Figure 2. Cryo-EM density map. Representative areas of the density map around protein a P1; b P2; c P3; d P4; e P5; f P6; g P7; h P8; i P9; j P10; k P11; l P12; m P13 and n P14. o The two P10 molecules within an icosahedral asymmetric unit.


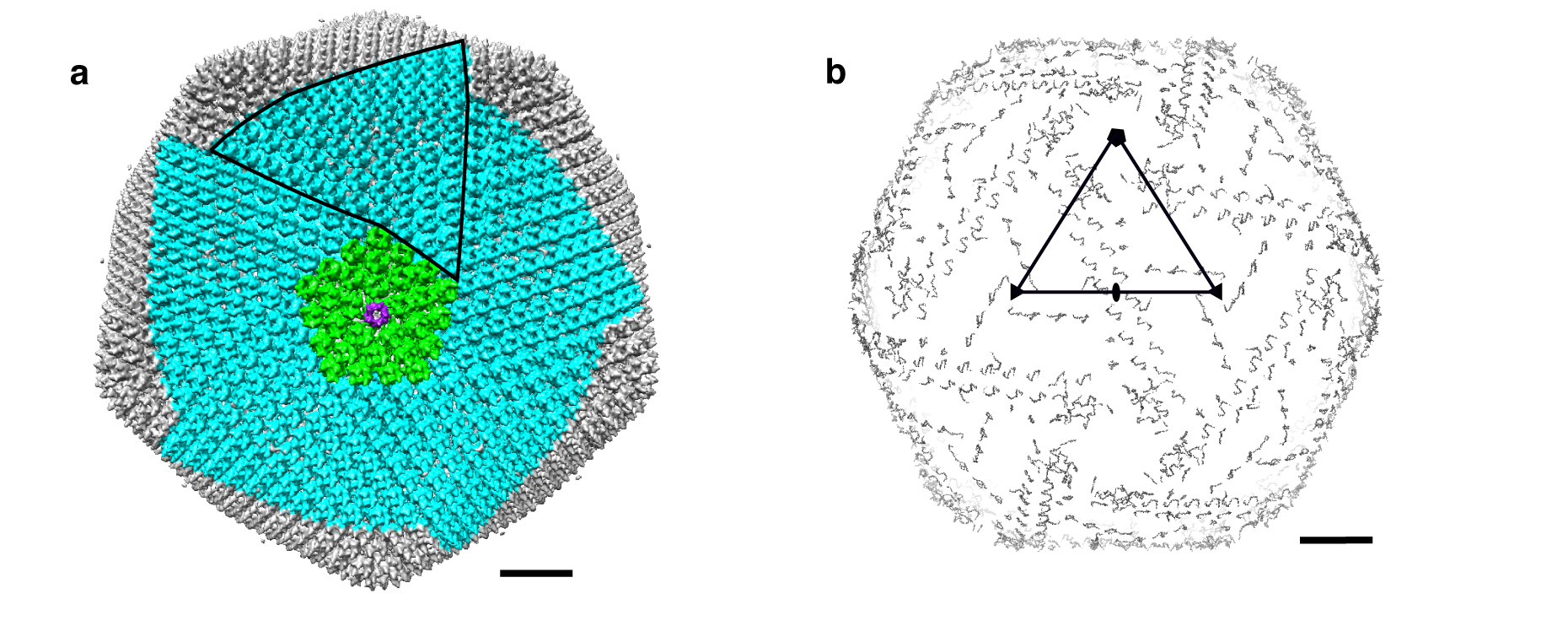


Supplementary Figure 3. Symmetrons and the uninterpreted densities of the icosahedrally averaged PBCV-1 map. a Schematic representation of the arrangement of trisymmetrons and pentasymmetrons in PBCV-1. The cryo-EM map of PBCV-1 has been low pass filtered to 15 Å resolution. The pseudo-hexameric capsomers in the pentasymmetron at one icosahedral 5-fold axis is shown in green, and the central pentameric capsomer is colored in purple. The trisymmetrons surrounding the pentasymmetron are shown in cyan. One trisymmetron is outlined in black. b The uninterpreted densities of the icosahedrally averaged PBCV-1 map. The icosahedral asymmetric unit of the map is shown as a black triangle, with the icosahedral 5-, 3-, and 2-fold axes shown as black pentagons, triangles and ovals, respectively. (Scale bars, 20 nm.)

**
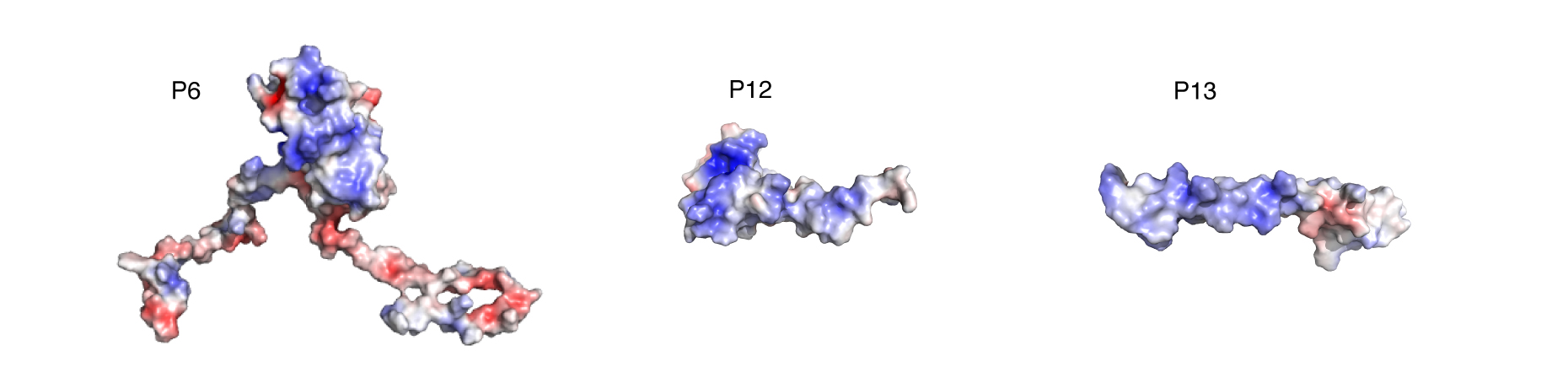
**

**Supplementary Figure 4.** The mostly positive charge distribution of the surfaces of P6, P12 and P13 minor capsid proteins that face the viral membrane. Blue and red colors correspond to 5 kTe^–^ positive and negative potential, respectively.


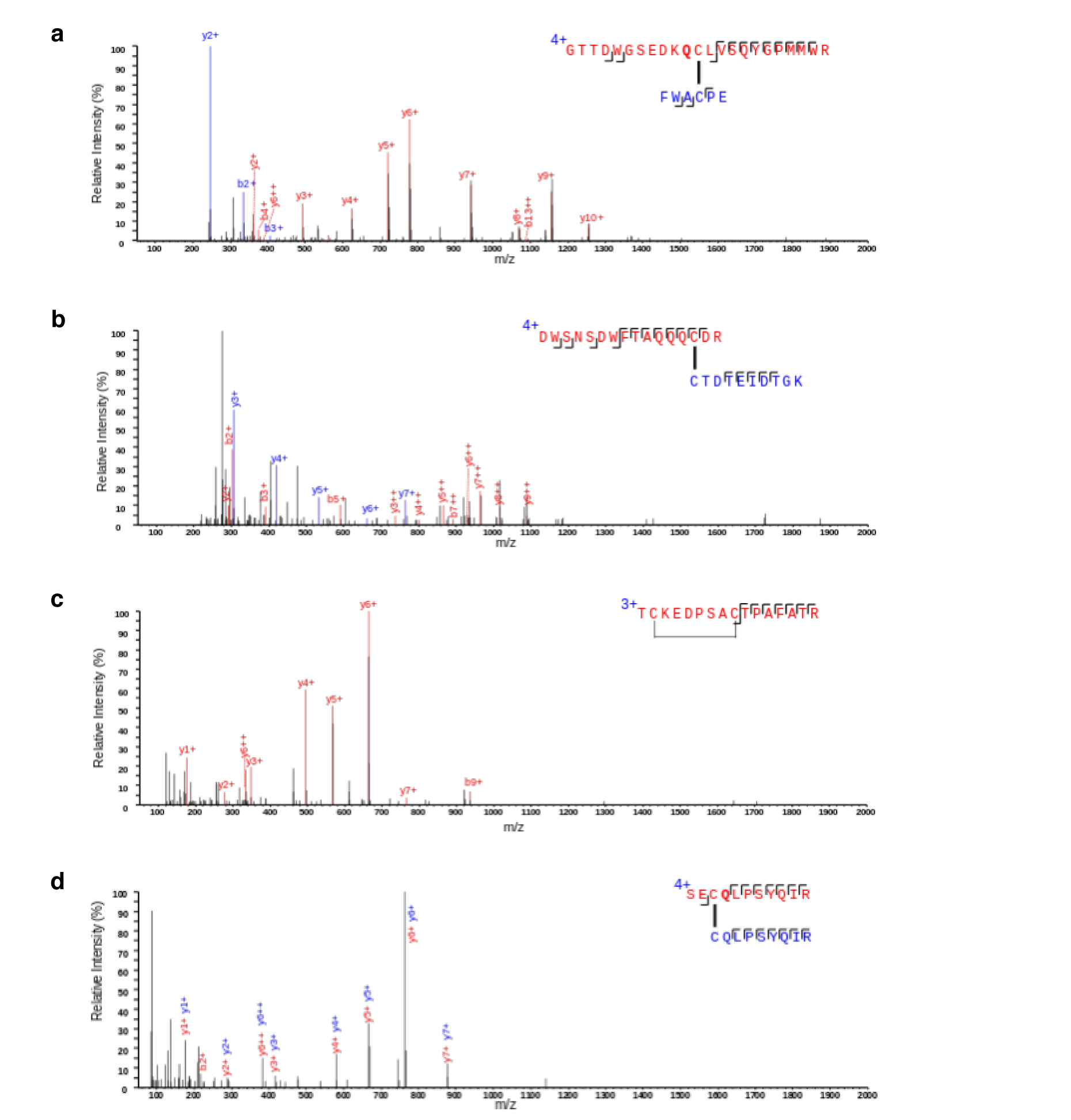


**Supplementary Figure 5.** Product-ion (MS/MS) spectra showing the identification of disulfide bonds using NEM labeling and plink search. The fragment y or b ions for each of the disulfide linked peptides are represented by red and blue colors, respectively. Spectra shown represent the disulfide bonds identified in P7 C120-C134 (a), C188-C211 (b), P9 C194-C201 (c) and P10 C215-C215 (d), respectively. The sequences of each peptides and the charge states are shown along with the figure. The glutamines Q119 (a) and Q216 (d) in bold indicate the presence of deamidation modification on that residue in each peptide.

Supplementary Table 1

Information on identified proteins

| **Protein name** | **Gene name** | **Number^a^** | **Protein sequence length** | **Ordered part** | **Top score^b^** | **Second highest score^c^** | **CC^d^** | **Resolution^e^ (FSC=0.25)** | **Resolution^e^ (FSC=0.5)** |
| --- | --- | --- | --- | --- | --- | --- | --- | --- | --- |
| **P1** | *a310l* | NA | 170 | 2-168 | 0.94 | NA | 0.77 | 3.6 | 4.0 |
| **P2** | *a342l* | NA | 576 | 94-568 | 0.89 | NA | 0.77 | 3.5 | 4.1 |
| **P3** | *a523r* | 1 | 171 | 27-167 | 0.87 | NA | 0.81 | 3.5 | 4.0 |
| **P3** | *a523r* | 2 | 171 | 22-168 | 0.86 | NA | 0.75 | 3.4 | 3.9 |
| **P3** | *a523r* | 3 | 171 | 49-164 | 0.87 | NA | 0.71 | 3.4 | 4.0 |
| **P3*** | *a523r* | NA | 171 | NA | 0.88 | NA | NA | NA | NA |
| **P4** | *a572r* | 1 | 181 | 5-62 | 0.89 | 0.72 | 0.82 | 3.5 | 3.8 |
| **P4** | *a572r* | 2 | 181 | 5-62 | 0.92 | 0.81 | 0.82 | 3.6 | 4.0 |
| **P4** | *a572r* | 3 | 181 | 5-107 | 0.9 | 0.8 | 0.79 | 3.5 | 3.9 |
| **P4** | *a572r* | 4 | 181 | 6-65 | 0.85 | 0.77 | 0.81 | 3.3 | 3.9 |
| **P4*** | *a572r* | NA | 181 | NA | 0.86 | 0.71 | NA | NA | NA |
| **P5** | *a526r* | NA | 146 | 4-145 | 0.9 | NA | 0.79 | 3.6 | 4.1 |
| **P6** | *a203r* | NA | 216 | 26-214 | 0.92 | NA | 0.77 | 3.6 | 3.9 |
| **P7** | *a262/263l* | NA | 256 | 98-254 | 0.91 | NA | 0.8 | 3.6 | 3.9 |
| **P8** | *a644r* | NA | 173 | 5-170 | 0.89 | NA | 0.81 | 3.6 | 4.0 |
| **P9** | *a407l* | NA | 210 | 102-208 | 0.92 | NA | 0.78 | 3.5 | 3.9 |
| **P10** | *a454l* | 1 | 289 | 192-285 | 0.85 | 0.62 | 0.78 | 3.6 | 3.9 |
| **P10** | *a454l* | 2 | 289 | 215-288 | 0.89 | 0.66 | 0.78 | 3.6 | 4.1 |
| **P10*** | *a454l* | NA | 289 | NA | 0.87 | 0.56 | NA | NA | NA |
| **P11** | *a352l* | 1 | 207 | 136-193 | NA | NA | 0.78 | 3.6 | 4.0 |
| **P11** | *a352l* | 2 | 207 | 153-206 | NA | NA | 0.81 | 3.6 | 4.1 |
| **P11** | *a352l* | 3 | 207 | 150-206 | NA | NA | 0.82 | 3.4 | 3.9 |
| **P11** | *a352l* | 4 | 207 | 154-205 | NA | NA | 0.79 | 3.5 | 4.0 |
| **P11** | *a352l* | 5 | 207 | 149-205 | NA | NA | 0.76 | 3.3 | 4.2 |
| **P11** | *a352l* | 6 | 207 | 155-206 | NA | NA | 0.74 | 3.5 | 4.1 |
| **P11** | *a352l* | 7 | 207 | 155-205 | NA | NA | 0.82 | 3.5 | 3.9 |
| **P11** | *a352l* | 8 | 207 | 155-205 | NA | NA | 0.82 | 3.6 | 4.0 |
| **P11** | *a352l* | 9 | 207 | 155-205 | NA | NA | 0.8 | 3.5 | 3.8 |
| **P11** | *a352l* | 10 | 207 | 155-205 | NA | NA | 0.82 | 3.5 | 3.9 |
| **P11** | *a352l* | 11 | 207 | 160-206 | NA | NA | 0.83 | 3.6 | 3.9 |
| **P11** | *a352l* | 12 | 207 | 147-205 | NA | NA | 0.83 | 3.5 | 3.9 |
| **P11*** | *a352l* | NA | 207 | NA | 0.94 | NA | NA | NA | NA |
| **P12** | *a139l* | NA | 151 | 73-150 | 0.93 | 0.29 | 0.83 | 3.5 | 3.8 |
| **P13** | *a421r* | NA | 98 | 32-97 | 0.93 | 0.66 | 0.76 | 3.5 | 4.1 |
| **P14** | *a500l* | NA | 352 | 5-101 | 0.93 | 0.56 | 0.78 | 3.5 | 3.9 |

^*^For those proteins which have more than one copy within each icosahedral asymmetric unit, a final search was performed by combining density information from all the polypeptide chains of each protein.

^a^For those proteins which have more than one copy in each icosahedral asymmetric unit, a number 1, 2, 3 … has been assigned (see Fig. 1c).

^b^The matching score of the identified protein sequence calculated by the Python script (Methods).

^c^The second-highest matching score of other protein sequence candidates calculated by the Python script (Methods).

^d^Real-space correlation coefficient between the model and the density map.

^e^FSC between the model and the density map.

NA = not applicable.

Supplementary Table 2

Potential disulfide bonds of identified proteins

| Protein name | Intramolecular disulfide bonds^a^ | Intermolecular disulfide bonds^a^ |
| --- | --- | --- |
| P1 | None | None |
| P2 | None | None |
| P3 | None | None |
| P4 | None | None |
| P5 | None | None |
| P6 | None | None |
| P7 | C120-C134, C156-C172, C188-C211 | None |
| P8 | None | None |
| P9 | C104- 131, C194-C201 | None |
| P10 | None | C215 (P10)-C215(P10) |
| P11 | None | None |
| P12 | C112-C120 | None |
| P13 | C82-C90 | None |
| P14 | C9-C101 | None |

^a^Structurally observed potential disulfide bonds with those confirmed by mass spectrometry colored in red.

Supplementary Table 3

Potential transmembrane helices of the minor capsid proteins

| Protein name | Predicted transmembrane helices^a^ | Ordered part | Linker length^b^ |
| --- | --- | --- | --- |
| P2 | Residues 12-36 | Residues 94-568 | 57 a.a. |
| P6 | Residues 2-20 | Residues 26-214 | 5 a.a. |
| P7 | Residues 24-67 | Residues 98-254 | 30 a.a. |
| P9 | Residues 58-80 | Residues 102-208 | 21 a.a. |
| P10 | Residues 2-20 | Residues 192-288 | 171 a.a. |
| P11 | Residues 6-23 | Residues 136-206 | 112 a.a. |
| P12 | Residues 20-42, 50-67 | Residues 73-150 | 5 a.a. |
| P13 | Residues 4-23 | Residues 32-97 | 8 a.a. |
| P14 | Residues 243-265 | Residues 5-101 | 142 a.a. |

^a^Transmembrane helices were predicted by TMHMM^4,5^, HMMTOP^6,7^ and Phobius^8^. Only results that are consistent in at least two out of the three programs are shown.

^b^The linker length between the predicted transmembrane helices and the ordered part of each protein.

Supplementary Table 4

Cryo-EM data collection, refinement and validation statistics

|  | #1 PBCV-1  (EMDB-0436)  (PDB 6NCL) |
| --- | --- |
| **Data collection and processing** |  |
| Magnification | 18,000 |
| Voltage (kV) | 300 |
| Electron exposure (e^–^/Å^2^) | 24 |
| Defocus range (μm) | 1.0-4.0 |
| Pixel size (Å) | 1.62 (physical pixel size) |
| Symmetry imposed | I4 |
| Initial particle images (no.) | 13,807 |
| Final particle images (no.) | 13,000 |
| Map resolution (Å)  FSC threshold | 3.5  0.143 |
| Map resolution range (Å) | 3.5-4.7 |
|  |  |
| **Refinement** |  |
| Initial model used (PDB code) | 5TIP |
| Map sharpening B factor (Å^2^) | -145 |
| Model composition  Non-hydrogen atoms  Protein residues  Ligands | 305,842  39,649  0 |
| *B* factors (Å^2^)  Protein  Ligand | 50 |
| R.m.s. deviations  Bond lengths (Å)  Bond angles (°) | 0.018  1.730 |
| Validation  MolProbity score  Clashscore  Poor rotamers (%) | 1.71  10.16  0.21 |
| Ramachandran plot  Favored (%)  Allowed (%)  Disallowed (%) | 96.95  2.89  0.16 |

Supplementary Table 5

Score of amino acid identification for sequence alignment

| **Size^a^** | **0**  **(G)** | **1**  **(A, C, P, S)** | **2**  **(T, V)** | **3**  **(D, I, L, N)** | **4**  **(E, H, K, M, Q)** | **5**  **(F, R, Y)** | **6**  **(W)** |
| --- | --- | --- | --- | --- | --- | --- | --- |
| **0** | 1.00 | 0.83 | 0.67 | 0.50 | 0.33 | 0.17 | 0.00 |
| **1** | 0.00 | 1.00 | 0.83 | 0.67 | 0.50 | 0.33 | 0.17 |
| **2** | NA | 0.00 | 1.00 | 0.83 | 0.67 | 0.50 | 0.33 |
| **3** | NA | NA | 0.00 | 1.00 | 0.83 | 0.67 | 0.50 |
| **4** | NA | NA | NA | 0.00 | 1.00 | 0.83 | 0.67 |
| **5** | NA | NA | NA | NA | 0.00 | 1.00 | 0.83 |
| **6** | NA | NA | NA | NA | NA | 0.00 | 1.00 |

^a^Side-chain size number of amino acids in the potential protein sequences (row) or as estimated using the cryo-EM map (column).

**Supplementary** **Reference**

1 Guo, F. & Jiang, W. Single particle cryo-electron microscopy and 3-D reconstruction of viruses. *Methods Mol Biol* **1117**, 401-443 (2014).

2 Zhu, D. *et al.* Pushing the resolution limit by correcting the Ewald sphere effect in single-particle Cryo-EM reconstructions. *Nat Commun* **9**, 1552 (2018).

3 Kucukelbir, A., Sigworth, F. J. & Tagare, H. D. Quantifying the local resolution of cryo-EM density maps. *Nat Methods* **11**, 63-65 (2014).

4 Krogh, A., Larsson, B., von Heijne, G. & Sonnhammer, E. L. Predicting transmembrane protein topology with a hidden Markov model: application to complete genomes. *J Mol Biol* **305**, 567-580 (2001).

5 Sonnhammer, E. L., von Heijne, G. & Krogh, A. A hidden Markov model for predicting transmembrane helices in protein sequences. *Proc Int Conf Intell Syst Mol Biol* **6**, 175-182 (1998).

6 Tusnady, G. E. & Simon, I. Principles governing amino acid composition of integral membrane proteins: application to topology prediction. *J Mol Biol* **283**, 489-506 (1998).

7 Tusnady, G. E. & Simon, I. The HMMTOP transmembrane topology prediction server. *Bioinformatics* **17**, 849-850 (2001).

8 Kall, L., Krogh, A. & Sonnhammer, E. L. A combined transmembrane topology and signal peptide prediction method. *J Mol Biol* **338**, 1027-1036 (2004).
